# The RabGAP TBC-11 controls Argonaute localization for proper microRNA function in *C*. *elegans*

**DOI:** 10.1371/journal.pgen.1009511

**Published:** 2021-04-07

**Authors:** Pascale Michaud, Vivek Nilesh Shah, Pauline Adjibade, Francois Houle, Miguel Quévillon Huberdeau, Rachel Rioux, Camille Lavoie-Ouellet, Weifeng Gu, Rachid Mazroui, Martin J. Simard

**Affiliations:** 1 CHU de Québec-Université Laval Research Center (Oncology division), Québec, Canada; 2 Université Laval Cancer Research Centre, Québec, Québec, Canada; 3 Department of Molecular, Cell and Systems Biology, University of California Riverside, Riverside, California, United States of America; University of Cambridge, UNITED KINGDOM

## Abstract

Once loaded onto Argonaute proteins, microRNAs form a silencing complex called miRISC that targets mostly the 3’UTR of mRNAs to silence their translation. How microRNAs are transported to and from their target mRNA remains poorly characterized. While some reports linked intracellular trafficking to microRNA activity, it is still unclear how these pathways coordinate for proper microRNA-mediated gene silencing and turnover. Through a forward genetic screen using *Caenorhabditis elegans*, we identified the RabGAP *tbc-11* as an important factor for the microRNA pathway. We show that TBC-11 acts mainly through the small GTPase RAB-6 and that its regulation is required for microRNA function. The absence of functional TBC-11 increases the pool of microRNA-unloaded Argonaute ALG-1 that is likely associated to endomembranes. Furthermore, in this condition, this pool of Argonaute accumulates in a perinuclear region and forms a high molecular weight complex. Altogether, our data suggest that the alteration of TBC-11 generates a fraction of ALG-1 that cannot bind to target mRNAs, leading to defective gene repression. Our results establish the importance of intracellular trafficking for microRNA function and demonstrate the involvement of a small GTPase and its GAP in proper Argonaute localization *in vivo*.

## Author summary

MicroRNAs play an essential contribution among the many mechanisms used by cells to maintain a proper gene expression program. These endogenous small RNAs regulate gene expression by binding to target mRNAs and blocking their translation into functional proteins. To achieve this, microRNAs rely on Argonaute and other proteins that form a complex called the miRISC. While research on microRNAs has brought a lot of interest over the last years, it is still unclear what modulators affect the activity of the miRISC. Our study identified proteins implicated in intracellular trafficking as important factors for miRISC function. We showed that the intracellular location of the Argonaute protein needs to be tightly regulated to assure proper microRNA-mediated mRNA repression in animals.

## Introduction

MicroRNAs (miRNA) are endogenous small RNAs that regulate a wide variety of biological functions through post-transcriptional gene silencing. Upon binding to Argonaute protein to form a functional complex termed microRNA-Induced Silencing Complex (miRISC), miRNAs guide the effector complex to a target mRNA, typically in the 3’ untranslated region (3’ UTR), through base pairing of its seed sequence (nt 2–8) [1,2]. Alternatively, some miRNAs bind mRNA targets in a non-canonical manner and rely on the 3’ half of the miRNA for binding [3,4]. With the help of effector proteins such as GW182, the miRISC can induce different molecular mechanisms leading to the abrogation of protein expression from the targeted mRNA such as translational repression, deadenylation, decapping or decay. While it is still unclear which one of these repression mechanisms occurs first, some studies suggest that the choice of favoured mechanism may be context specific (for example, see [5–11]). The composition and repression mechanism of miRISC therefore vary between cell types and stages of development.

Gene silencing by miRNA is known to occur in the cytoplasm of cells. However, the specific subcellular localization of miRNAs and their effector complex miRISC remains elusive. Several reports have linked miRNA function to endomembrane trafficking. Some studies have shown that miRISC components’ association with multivesicular bodies (MVBs) is important for miRNA function both in mammalian cells and in *Drosophila* [12,13]. Interestingly, some studies have linked small RNA function to the Endoplasmic Reticulum (ER) and the Golgi apparatus. Forward genetic screens in *C*. *elegans* revealed that proteins associated with the Golgi apparatus are involved in miRNA-mediated gene regulation. For example, *cogc-4*, a member of the oligomeric Golgi complex, was reported to be involved in the miRNA pathway [14]. In addition, components of the Golgi Associated Retrograde Protein (GARP) complex were also identified as important factors for miRNA function [15].

A study in mammalian cells has shown that the siRNA and miRNA loading onto Argonaute, as well as target repression, occur at the rough ER [16]. Furthermore, studies in *Arabidopsis thaliana* also showed that miRNAs associate with membrane-bound polysomes in an Argonaute-dependent manner and that target repression occurs at the ER [17,18]. Other studies in mammalian cells suggested that miRNA targets need to be sent to the rough ER before repression can occur [19], and that Argonaute proteins are transported to the ER to be loaded with miRNA [20]. Although some studies identified the ER as a site of target repression, they could not exclude that other targets may be repressed at different cellular locations than the ER. This is well represented in neurons, where specific pre-miRNAs are transported in the dendrites to be processed as miRNA and repress locally mRNA targets to regulate dendritic development [21–23]. Moreover, this transport of pre-miRNAs to dendrites is mediated by late endosomes and lysosomes [24]. Altogether, these studies highlight the importance of the transport of miRNAs and miRISC components within the cell. However, while many reports point towards an important role for endomembrane trafficking in miRNA function, it is still unclear how these two mechanisms are related and how miRISC components are transported between different compartments of the cell.

In this study, we identify *tbc-11*, a Rab GTPase activating protein, as a new important factor for proper miRISC function in *C*. *elegans*. We demonstrate that *tbc-11* is involved in miRNA-mediated gene regulation through mainly *rab-6*, a Golgi-associated small GTPase. We observed that misregulation of TBC-11 affects the localization of the miRNA-specific Argonaute ALG-1 as well as its association with miRNAs. This leads to an accumulation of miRNA-unloaded ALG-1 in a perinuclear, high molecular weight complex, inducing defects in miRISC target repression *in vivo*.

## Results

### The Rab GTPase activating protein TBC-11 contributes to miRNA function

**Fig 1:**
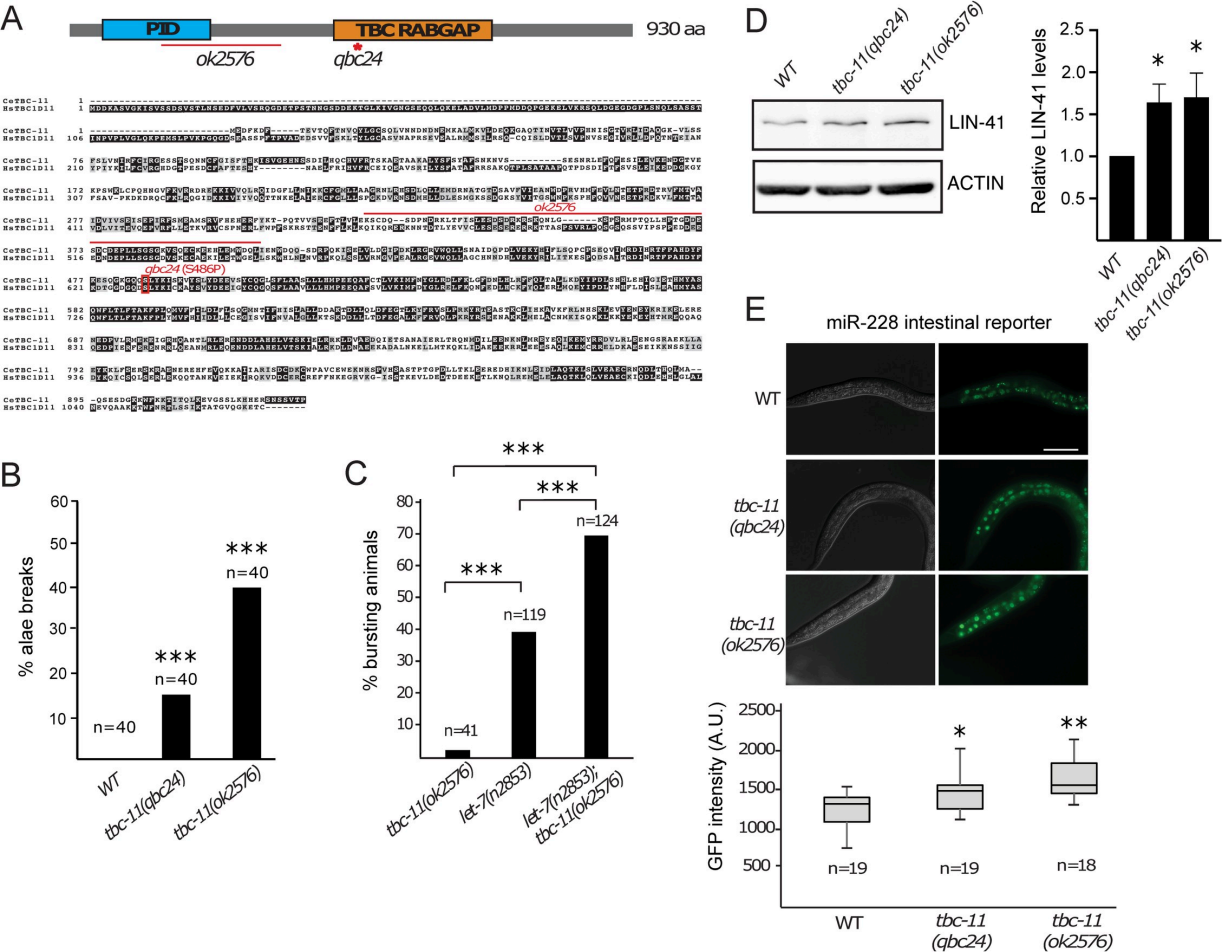
*tbc-11* is important for miRNA function. (**A**) Top: Schematic representation of TBC-11 protein domains with the two studied alleles. *ok2576* is a deletion allele (a 1421 base pairs deletion that span from the middle intron 9 to the middle exon 11) and *qbc24* allele is a missense mutation in the catalytic domain of the protein. PID: phosphotyrosine interaction domain. Bottom: ClustalW alignment of human (Hs) and *C*. *elegans* (Ce) protein sequence shows conservation between species that share 41.1% identity and 71.3% similarity. The amino acid mutated in *qbc24* allele is conserved in humans. (**B**) Alae breaks of *tbc-11(qbc24)* and *tbc-11(ok2576)* young adult animals were scored under DIC Nomarski microscopy. The number of animals scored (n) is indicated. P value were obtained by two tailed t-test (***p value < 0.0001). (**C**) Percentage of animals bursting through the vulva were scored at 15°C. The number of animals scored (n) is indicated. *P* value were obtained by one-way ANOVA (***p value < 0.0001). (**D**) Western blot of LIN-41 in wild-type (WT), *tbc-11(qbc24)* and *tbc-11(ok2576)* young adult worm extracts (left). Actin is used as a loading control. Quantification of 4 independent Western blots is shown (right). LIN-41 level is normalized to Actin levels. *P* value were obtained by one-way ANOVA (*p value < 0.05). (**E**) Top: DIC and fluorescent microscopy of miR-228 activity reporter in intestine cells of L2 staged animals. Scale bar: 50μm. Bottom: Quantification of GFP fluorescence intensity in three intestine cells per animal. Quantification was performed by measuring GFP intensity in a consistent-sized circle that was drawn around each intestine cell nucleus. Images were taken using the same settings and exposition time for each animal. The number of animals scored (n) is indicated. *P* value were obtained by two tailed t-test (*p value < 0.05, **p value < 0.001).

We performed a forward genetic screen in *C*. *elegans* to identify new factors important for miRISC silencing. To focus on factors involved in miRISC function at the mRNA target level, we took advantage of the λN/Box B tethering system to recruit a functional λN tagged-ALG-1 protein to a GFP transgene in which 6 Box B sequences were inserted in the 3’ UTR (as described in [9]; S1A Fig). In this condition, the recruitment of the miRISC-specific Argonaute ALG-1 to the reporter mRNA causes the repression of the GFP expression and mutations that disrupt miRISC function would be expected to alleviate this repression, leading to stronger GFP expression. Following chemical mutagenesis, we identified a mutant allele *(qbc24)* in which GFP repression was altered (S1B Fig). After genetic mapping that located the causal mutation on chromosome X and high-throughput genome sequencing of extensively outcrossed mutant animals, we identified the only missense/nonsense mutation on the chromosome X of *qbc24* animal in the coding sequence of the *tbc-11* gene (Fig 1A). To test if the *qbc24* allele represents a dominant allele of *tbc-11*, we monitored the alae structure of heterozygote animals. As *qbc24*/+ animals did not display any defects (S1C Fig), we conclude that the *qbc24* allele is a recessive allele.

TBC-11 is a conserved Rab GTPase activating protein (RabGAP) that acts on the Rab family of small GTPases. Rab proteins are implicated in all steps of vesicular trafficking and alternate between an active GTP-bound and inactive GDP-bound state (reviewed in [25,26]). GAPs such as TBC-11 catalyze the hydrolysis of GTP to GDP, therefore inactivating their targeted Rab. In addition to our isolated mutant *tbc-11(qbc24)*, in which a conserved serine residue within the catalytic domain is changed for a proline, a deletion allele *tbc-11(ok2576)* was also available and further used for characterization (Fig 1A).

To test whether *tbc-11* is involved in the miRNA pathway, we first looked at the animals’ alae formation. This cuticular structure of the worm is formed by the asymmetric divisions of seam cells throughout larval development followed by their fusion at the L4 to adult transition to form the alae. These programmed divisions of seam cells are tightly controlled by miRNA lin-4 and the let-7 miRNA family [27–29]. Disruption of genes associated to the miRNA pathway, such as *alg-1*, induces extra divisions of seam cells that leads to discontinuities in the alae structure (termed breaks) [30]. These defects have also been observed for many modulators of miRNA function (for example, see [15,31–33]). We, therefore, scored the number of seam cells in both alleles of *tbc-11* to assess their importance in this miRNA-regulated process. While wild-type animals have an invariable number of 16 seam cells at the adult stage, we observed a range in the number of seam cells in both *tbc-11* mutant animals (S1D Fig). When we surveyed the alae structures of *tbc-11* mutants, we observed the presence of breaks in both alleles, indicating that *tbc-11* is important for the normal seam cell division pattern controlled by miRNAs (Fig 1B).

To determine if *tbc-11* is involved more broadly in the regulation by the let-7 family miRNAs, we tested its synthetic effect with a *let-7* mutant allele. This sensitized genetic background allows to monitor the involvement of various factors in the miRNA pathway as it presents partial penetrance on its own. The complete loss of let-7 miRNA induces a distinctive phenotype of bursting through the vulva [29]. The temperature-sensitive *let-7(n2853)* allele is completely penetrant at the restrictive temperature of 25°C but only partially penetrant at the permissive temperature of 15°C [29]. We thus used this mutant allele to test the involvement of *tbc-11(ok2576)* mutation on the bursting of *let-7(n2853)* animals. We could not assess the involvement of *tbc-11(qbc24)* allele on *let-7(n2853)* since both animals produce sterile males. Although *tbc-11(ok2576)* mutant population have few bursting animals, we observed an important increase in bursting in *tbc-11(ok2576); let-7(n2853)* compared to *let-7(n2853)* animals alone (Fig 1C). Together with the defects observed in the division pattern of seam cells and the alae structure, our data suggest that *tbc-11* is implicated in the developmental regulation by the let-7 family miRNAs.

We next wanted to assess whether the defects observed in the regulation by *let-7* would alter the levels of its well-established target, LIN-41. To test this, we first used a GFP reporter fused to the *lin-41* 3’ UTR region and measured the repression by *let-7* in L4-staged animals, as *let-7* is only expressed in late larval stages [29, 34]. We observed a significant derepression of GFP upon depletion of *tbc-11* (RNAi), suggesting that *tbc-11* is important for repression of a reporter gene under the control of *lin-41* 3’UTR (S1E Fig). To further confirm that *tbc-11* contributes to the regulation of LIN-41 protein in animals, we monitored the levels of endogenous LIN-41 expression by western blot in *tbc-11* animals. We observed that the alteration of TBC-11 found in both *tbc-11(qbc24)* and *tbc-11(ok2576)* adult animals leads to a significant increase of LIN-41 protein compared to wild-type animal extracts (Fig 1D), confirming the implication of *tbc-11* in the regulation of the microRNA target LIN-41.

To determine if *tbc-11* affects miRNAs other than the let-7 family, we tested its contribution on the repression of a miR-228-dependent reporter [7]. While miR-228 represses the GFP reporter expressed in the intestine cells in wild-type animals, the alteration of *tbc-11* leads to a significant derepression in both alleles (Fig 1E), indicating that *tbc-11* is required for proper repression by miR-228. To confirm that this derepression was miR-228 dependent, we monitored if *tbc-11* alleles had an effect on a reporter in which miR-228 binding sites have been removed. We observe no significant differences in GFP expression with this reporter (S1F Fig), confirming that the effect observed is miRNA-dependent. We therefore conclude that *tbc-11* is important for the function of miRNAs in different tissues of the animal.

Altogether, these results suggest that *tbc-11* is involved in miRNA function and that its loss induces repression defects for various miRNAs.

### TBC-11 acts mainly on RAB-6 to affect miRNA function

**Fig 2:**
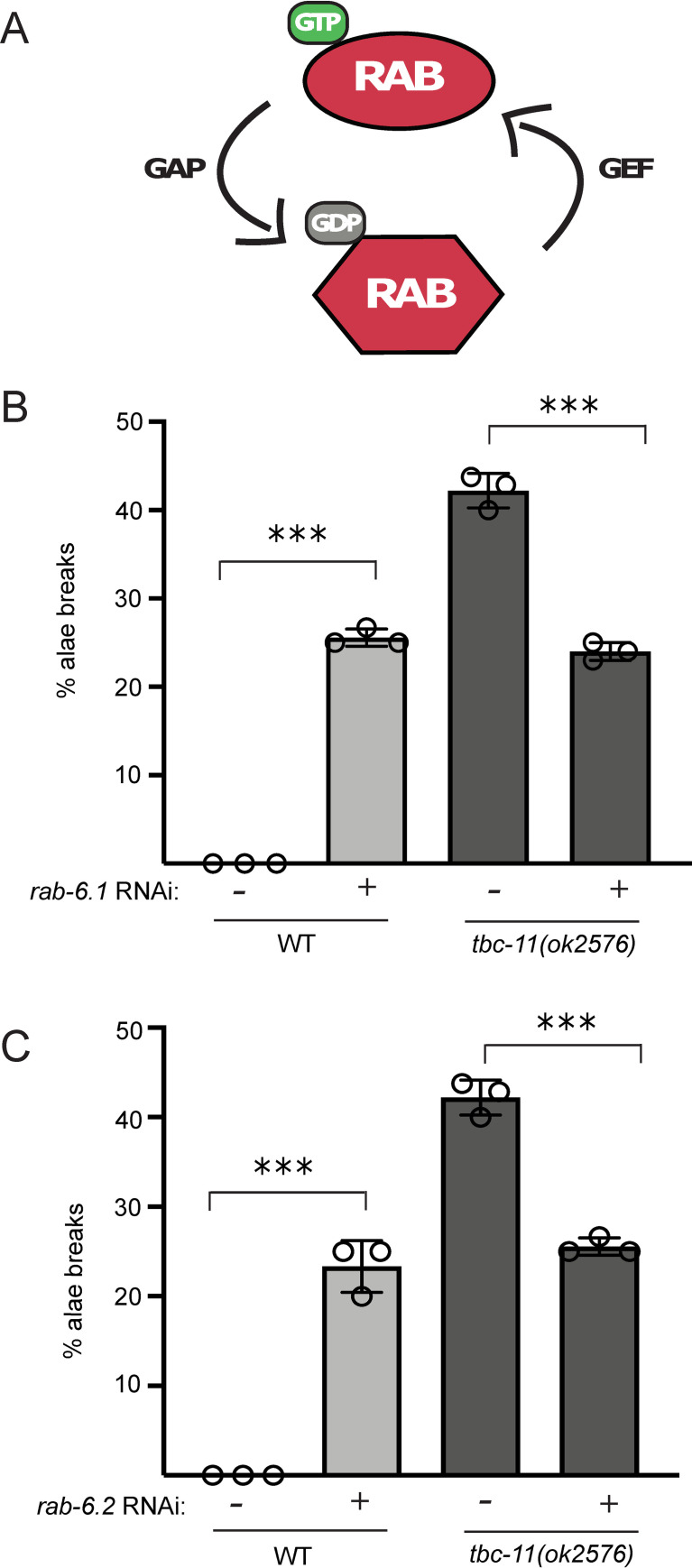
TBC-11 is likely a GTPase activating protein for RAB-6.1 and RAB-6.2. (**A**) The relationship between Rab GTPase, GAPs and GEFs. Rab proteins are active when bound to GTP and inactive when bound to GDP. Guanine nucleotide exchange factors (GEF) activate Rab by releasing the bound GDP molecule to allow the binding of a GTP molecule. GTPase activating proteins (GAP) catalyze the hydrolysis of GTP to GDP to inactivate Rab. (**B**-**C**) Alae breaks of *tbc-11(ok2576)* were scored under DIC Nomarski microscopy. Animals were fed with bacteria expressing RNAi against *rab-6*.*1*, *rab-6*.*2* or control RNAi (no targeting gene) for 48 hours and observed as young adults. 50 animals were observed for each condition. Each circle represents the mean of one independent RNAi experiment. *P* value were obtained by one-way ANOVA (***p value < 0.0001).

Rab proteins need to alternate between an active and inactive state to regulate vesicular trafficking (Reviewed in [25,26]). Guanine nucleotide exchange factors (GEF) are implicated in activation of the Rab by releasing the bound GDP to allow binding of a GTP molecule. GTPase activating proteins (GAP) are important for hydrolysis of GTP to GDP, therefore inactivating their targeted Rab (Fig 2A). This cycle can then be repeated at every round of vesicular transport. Since distinct Rabs are associated with different steps of vesicular transports, we wanted to know which Rab was targeted by *tbc-11* to regulate miRNA function. As the absence of a GAP (such as TBC-11) will lead to a constitutive activation of the targeted Rab, we expect that reducing its expression will rescue the phenotypes observed in the GAP mutant animals. In *C*. *elegans*, TBC-11 had never been characterized as a GAP for any Rab protein. Its human ortholog, TBC1D11, was however known to be a GAP for human Rab2, Rab4, Rab6, Rab11 and Rab14 (Reviewed in [25]). While Rab2, Rab4 and Rab11 can be deactivated by more than one GAP, Rab6 seems like a better candidate to test since TBC1D11 was its only known GAP. Moreover, Rab6 is known to localize at the Golgi apparatus and interact with the GARP complex, which was previously shown to be important for miRNA function [15]. We therefore tested first if TBC-11 could be a GAP for the two Rab6 orthologs RAB-6.1 and RAB-6.2 in *C*. *elegans*. We scored alae breaks in *tbc-11(ok2576)* and observed around 40% of animals with breaks. When exposed to RNAi against *rab-6*.*1* or *rab-6*.*2*, we observed that alae breaks of *tbc-11(ok2576)* animals were suppressed, indicating that these defects were most likely caused by a persistent activation of RAB-6 (Fig 2B and 2C). We further tested if alae breaks in *tbc-11(ok2576)* animals could be suppressed by RNAi against other predicted Rab targets of *tbc-11*. We exposed WT and *tbc-11(ok2576)* animals to bacteria expressing dsRNA targeting *rab-2* and *rab-14* (the *C*. *elegans* ortholog of Rab4 and Rab14) and scored alae breaks (of note: RNAi against *rab-11*.*1* and *rab-11*.*2*, the *C*. *elegans* ortholog of Rab11, caused lethality in animals, and thus alae structure could not be assessed). While both RNAi induce alae breaks in WT animals (suggesting a potential contribution to the miRNA pathway), they did not suppress the alae breaks in *tbc-11(ok2576)* animals at the same level as observed for *rab-6*.*1* and *rab-6*.*2* (S2 Fig). Based on these results and on the conservation of RAB and GAP proteins across species, we can conclude that RabGAP TBC-11 acts mainly on or through the small GTPase RAB-6.1 and RAB-6.2 (and is likely a GAP for these proteins) to control miRNA function in animals.

### The RabGAP TBC-11 modulates the cellular distribution of ALG-1

**Fig 3:**
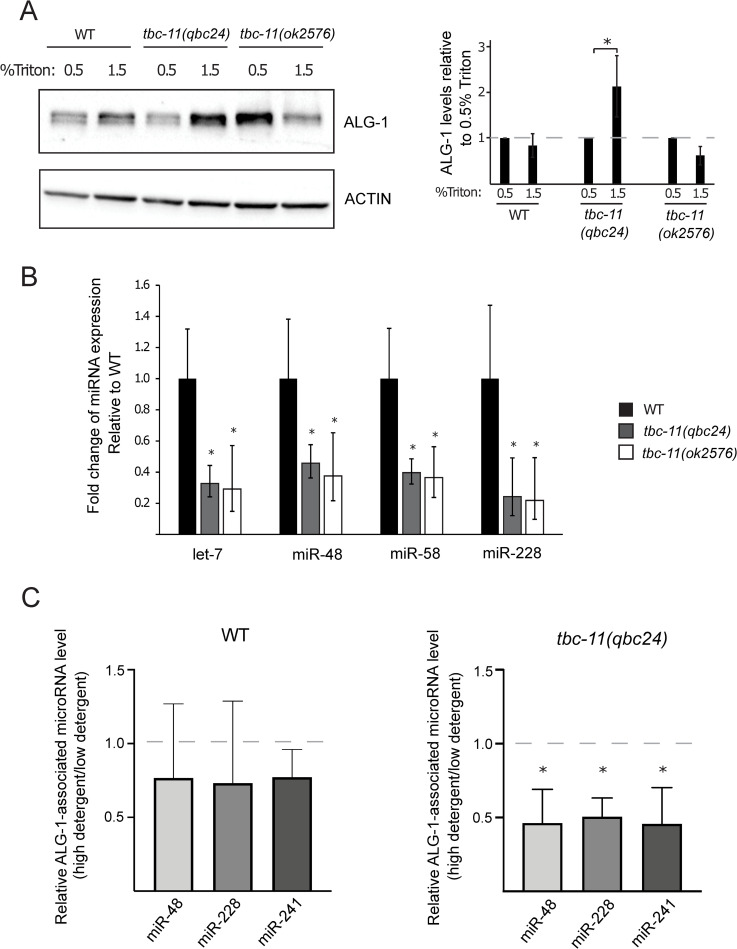
*tbc-11* affects ALG-1 association with endomembrane and microRNAs. (**A**) Western blot of ALG-1 in wild-type (WT), *tbc-11(qbc24)* and *tbc-11(ok2576)* young adult worm extracts prepared with low (0.5%) or high (1.5%) detergent (Triton) concentration. High detergent concentration allows better extraction of membrane associated proteins. Actin is used as a loading control. Quantification of 3 independent Western blots is shown. ALG-1 level is normalized to Actin levels and normalized to 0.5% detergent for each condition. *P* value were obtained by one-way ANOVA (*p value < 0.05). (**B**) Quantification of microRNA expression. The levels of let-7, miR-48, miR-58 and miR-228 were measured by RT-qPCR in *tbc-11* mutants and normalized on the levels in wild-type animals (n = 3). Small nucleolar RNA sn2841 was used as a reference. The error bars indicate the confidence interval (α = 0.05) and *P* values were calculated with a two-tailed Student t-test. (*p value < 0.05). **(C)** Expression level of ALG-1 associated miRNAs measured by RT-qPCR. RNA was extracted following ALG-1 immunoprecipitation in wild-type (WT) and *tbc-11(qbc24)* young adult worm extracts prepared with low (0.5%) or high (1.5%) detergent concentration. Ct values obtained for low detergent extracts were subtracted from Ct values obtained for high detergent extracts and log2 values were calculated. The Ct ratio was then normalized to the levels of immunoprecipitated ALG-1 (see S3E Fig for representative western blot) for each individual microRNA. Error bars represent standard deviation for each microRNA. *P* values were obtained by two tailed t-test (*p value < 0.05). Three independent immunoprecipitation experiments were performed.

RAB-6 is known to be located at the Golgi apparatus and is involved in many steps of trafficking such as endosome to Golgi, Golgi to plasma membrane, intra-Golgi as well as Golgi to ER (reviewed in [35]). Since we have shown that *tbc-11* affects miRNA function through *rab-6*, we next asked if the alteration of *tbc-11* could affect the localization of miRISC components to endomembranes. We first monitored the association of ALG-1 to the endomembranes by changing the level of detergent agents used during protein extracts preparations as detergent facilitates the extraction of membrane-associated proteins. We observed that in standard extraction conditions containing a low concentration of Triton (0.5%), protein extracts prepared from *tbc-11*(*qbc24)* animals had lower ALG-1 levels compared to WT, whereas *tbc-11*(*ok2576)* animals had higher ALG-1 levels in their extracts (S3A Fig). When the extracts were prepared with lysis buffer containing a higher concentration of detergent (1.5% Triton), which helps solubilize endomembrane-associated proteins (as well as aggregation-prone proteins), we observed a significant increase in ALG-1 levels in *tbc-11*(*qbc24)* extracts and a decrease in *tbc-11*(*ok2576)* (Fig 3A). This difference in ALG-1 solubility might be explained by the nature of the *tbc-11* mutations found in those alleles. The *ok2576* allele carries a large deletion of 1421 base pairs that most likely corresponds to a loss of function of *tbc-11* while the *qbc24* allele corresponds to a point mutation in the catalytic domain of the gene, which may act as a catalytic dead RabGAP and thus have a different molecular outcome on Rab activation. Based on those observations, we hypothesize that in *tbc-11(qbc24)* animals, ALG-1 may be more associated to endomembranes, whereas it would be less associated to endomembranes in *tbc-11*(*ok2576)* animals. We next wondered if this effect was widespread to the other miRISC components. We monitored the levels of the GW182 ortholog AIN-1 and observed that while the protein levels are affected in *tbc-11* mutants, this was not dependent on detergent concentration (S3B Fig), indicating that *tbc-11* acts specifically on ALG-1. To confirm that the differences observed in ALG-1 were not caused by changes in the total ALG-1 protein level, we performed western blots on fully solubilized extracts and observed no substantial differences in ALG-1 levels (S3C Fig). This result indicates that the differences observed in ALG-1 levels in the prepared extracts are likely caused by the ability to solubilize and recover membrane-associated or aggregated proteins.

### TBC-11 affects the association of ALG-1 to microRNAs

We next wanted to determine if the possible changes in ALG-1 endomembrane association altered miRNA levels as well as the loading of miRNAs onto the Argonaute, as these two properties are tightly linked [36]. We first monitored the total levels of a subset of mature miRNAs, including some let-7 family members, by RT-qPCR. We observed a significant decrease in all of the tested miRNA levels in both *tbc-11* mutant alleles (Fig 3B). We then performed high-throughput sequencing analysis of total small RNAs isolated from young adult animal population to determine whether the alteration of *tbc-11* has a global effect on miRNA levels. Even if the changes in total miRNA levels between wild-type and *tbc-11* animals monitored by this method are not statistically significant (S3D Fig), we can observe a decrease in most miRNA levels in *tbc-11* animals inferring that the loss of *tbc-11* could have a general impact on miRNA levels in animals.

We next wanted to determine if the association of ALG-1 to endomembranes or to an aggregated complex altered its ability to bind miRNAs. To answer this, we immunoprecipitated ALG-1 in extracts from wild-type and *tbc-11(qbc24)* animals, prepared with low or high detergent concentration, and monitored miRNAs associated to ALG-1 in both conditions. We observed that in wild-type animals, the concentration of detergent used in the extracts does not alter the level of miRNAs loaded into ALG-1 (Fig 3C; left). However, in *tbc-11(qbc24)* animals, the level of miRNAs loaded onto ALG-1 is significantly decreased when extracts are prepared with high detergent concentration (Fig 3C; right). This data suggests that the pool of ALG-1 that accumulates on endomembranes or in aggregated complexes in *qbc24* animals is not functional for gene silencing as it is not loaded with miRNAs. Moreover, this decrease in miRNA loading induces a decrease in total miRNA levels (as observed in Fig 3B) as these two properties are tightly linked.

### ALG-1 localizes at the perinuclear region and accumulates in a high molecular weight complex in *tbc-11(qbc24)* animals

**Fig 4:**
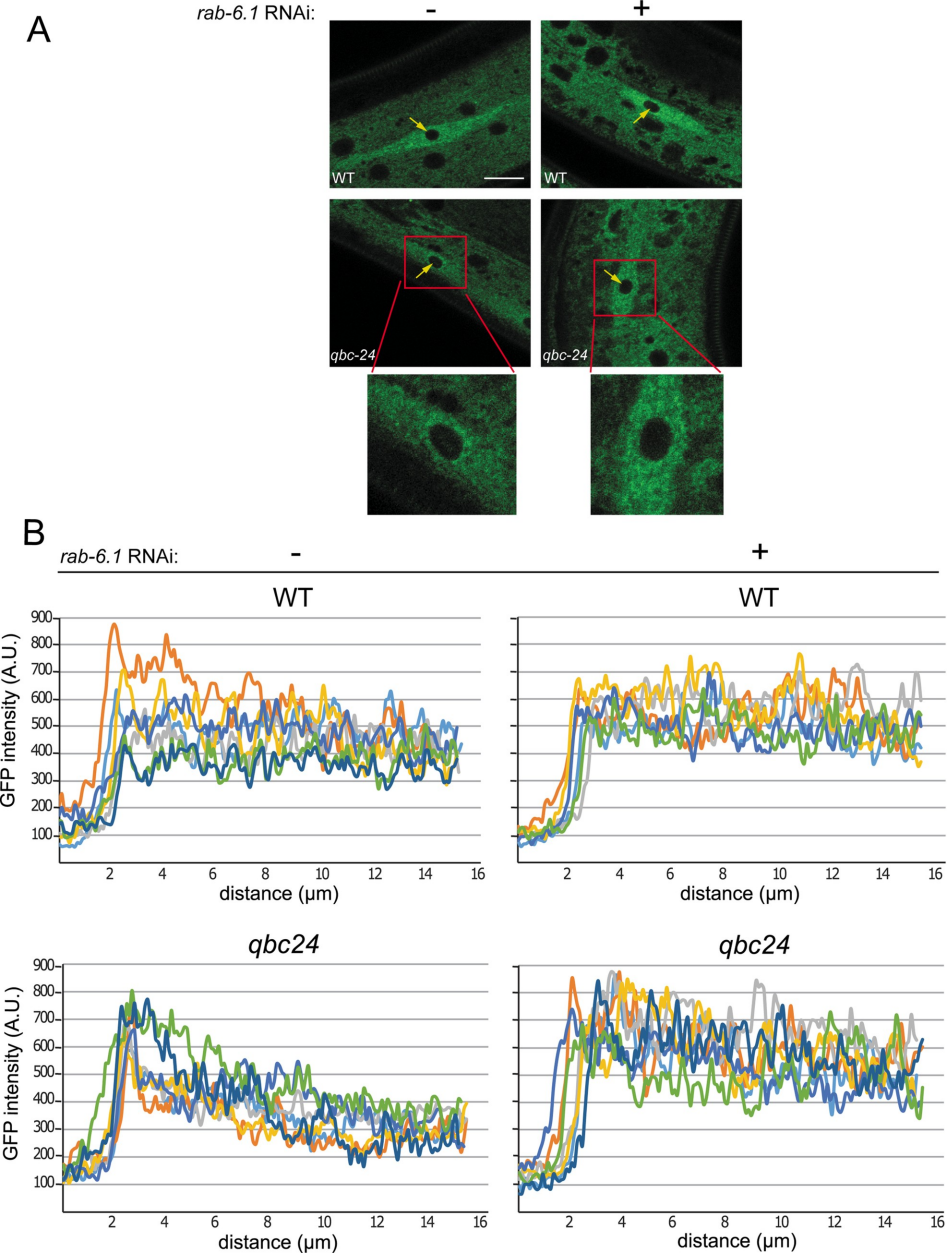
*tbc-11* affects ALG-1 intracellular localization. **(A)** Fluorescent microscopy of intracellular localization of endogenously tagged GFP::ALG-1 in seam cells of wild-type (WT) and *tbc-11(qbc24)* young adults animals. Animals were fed with bacteria expressing RNAi against *rab-6*.*1* or control RNAi (-;no targeting gene) for 48 hours. The nucleus of the seam cell is indicated by a yellow arrow. A zoomed in image of *tbc-11(qbc24)* seam cell is showed. Images were taken using the same settings and exposition time for each animal. Scale bar: 10μm. (**B**) Quantification of representative images of GFP::ALG-1 in seam cells of wild-type (WT) and *tbc-11(qbc24)* animals exposed to RNAi against *rab-6*.*1* or control RNAi (no targeting gene) for 48 hours. Quantification was performed by drawing a straight line starting from the middle of the nucleus and passing through the cytoplasm of the seam cell. The fluorescence signal was measured along the line using ImageJ. The distance in μm represents the distance from the middle of the nucleus. Each line represents the GFP quantification of a separate image of a different animal.

As we observed an accumulation of unloaded ALG-1 in *tbc-11(qbc24)* animals, we wondered if the subcellular localization of ALG-1 was altered. To test this, we observed the subcellular localization of an endogenous GFP-tagged ALG-1 in the seam cells of the animals. In wild-type animals, GFP::ALG-1 is localized broadly across the cytoplasm of the cell. However, in *tbc-11(qbc24)* animals, we observed that the pool of ALG-1 expressed in seam cells is mostly located around the nucleus (Fig 4). To confirm that this mislocalization of ALG-1 was dependent on RAB-6, we exposed *tbc-11* animals to *rab-6*.*1* RNAi and monitored ALG-1 localization. We observed that the subcellular localization of ALG-1 was not affected by *rab-6*.*1* RNAi alone (Fig 4). However, when *tbc-11* animals were exposed to *rab-6*.*1* RNAi, the localization of ALG-1 was comparable to wild type (i.e.; diffused in the cytoplasm), indicating that the mislocalization of ALG-1 observed in *tbc-11* is likely due to persistent activation of RAB-6 (Fig 4).

We have attempted to identify this perinuclear structure by expressing different organelle markers and observing co-localization with ALG-1. Since RAB-6 localizes at the Golgi, we first wondered if ALG-1 was accumulating at the Golgi in *tbc-11(qbc24)* mutants. We expressed the Golgi marker mannosidase::mCherry (MANS::mCherry), under the control of *alg-1* promoter in WT and *tbc-11(qbc24)* animals and monitored its localization pattern in the seam cells. While we observed a fraction of ALG-1 that co-localized with MANS::mCherry by microscopy, we did not observe an elevated amount of MANS around the nucleus as we observed for ALG-1 in *tbc-11(qbc24)* animals (S4 Fig). We next wondered if ALG-1 could be accumulating at the endoplasmic reticulum (ER) located at the periphery of the nucleus since RAB-6 is involved in Golgi to ER transport [35]. Many processes occur at the ER, including protein synthesis, protein folding, lipid synthesis and calcium storage. There are two distinct forms of ER: the rough ER defined by a high density of ribosomes involved mainly in protein synthesis and the smooth ER which has few ribosomes and is associated with lipid synthesis (reviewed in [37]). It was previously shown that Argonaute proteins associate with the rough ER for miRNA loading and mRNA target repression [16]. Moreover, many studies have shown that Argonaute associates with polysomes (for example, see [32,38–41]). We therefore hypothesized that the association of ALG-1 with the ribosomes at the perinuclear rough ER might be affected in the seam cells of *tbc-11(qbc24)* animals.

**Fig 5:**
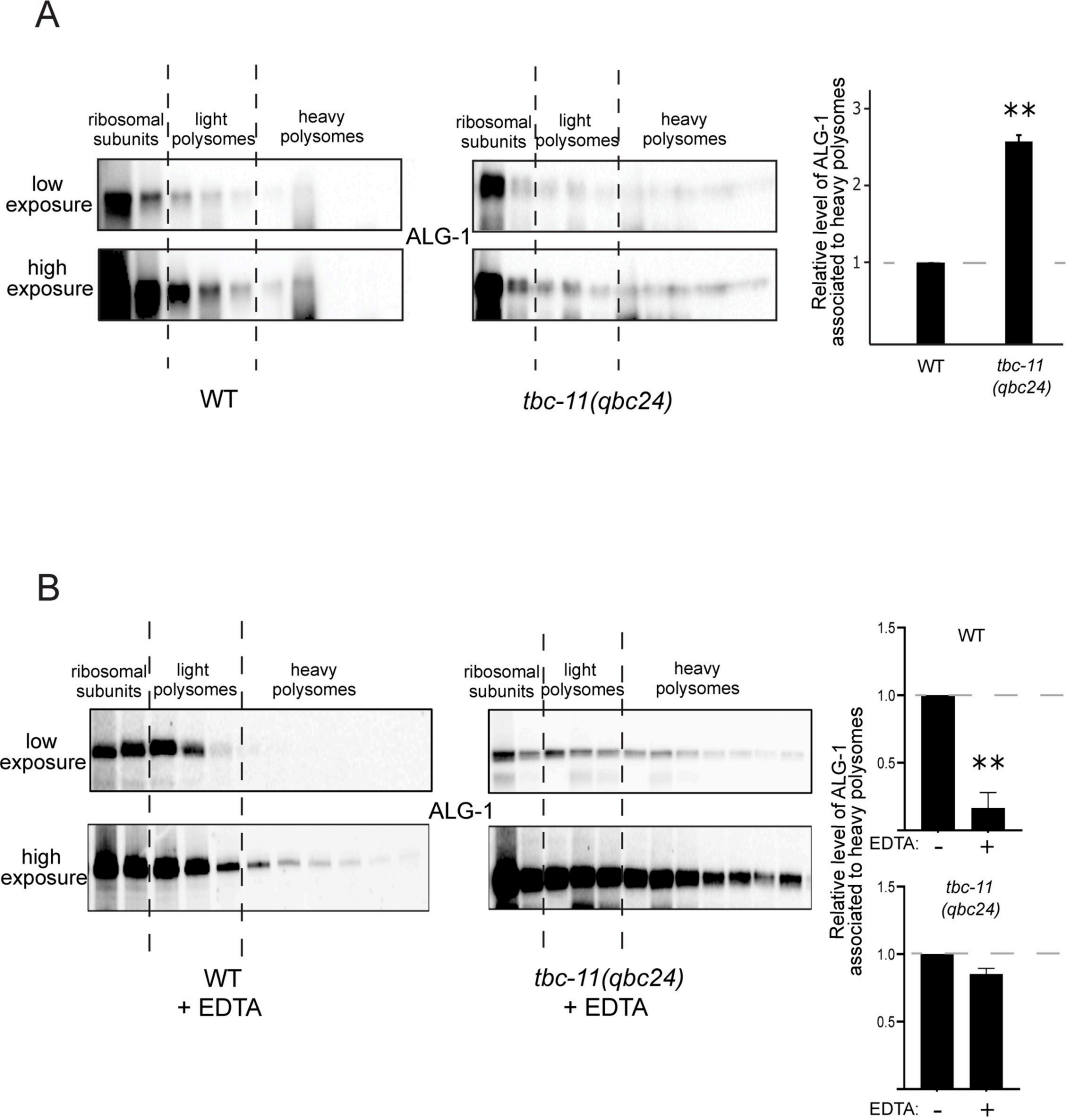
ALG-1 associates with a high molecular weight complex in *tbc-11(qbc24)* animals. **(A)** Detection of ALG-1 in different fractions by Western blot following polysome profiling of wild-type (WT) and *tbc-11(qbc24)* young adult animals performed with extracts prepared with high detergent concentration (1.5% triton). Low and high exposure of the same Western membrane are shown. Fractions corresponding to ribosomal subunits, light polysomes and heavy polysomes are indicated. (**B**) Detection of ALG-1 in different fractions by Western blot following polysome profiling of wild-type (WT) and *tbc-11(qbc24)* young adult animals performed with extracts treated with 10mM EDTA. Fractions corresponding to ribosomal subunits, light polysomes and heavy polysomes are indicated. Low and high exposure of the same Western membrane are shown. For both panels, the quantification of ALG-1 associated to heavy polysomes (to obtain the relative level of ALG-1 associated to heavy polysomes, the sum of the signal detected in those fractions was normalized to the total ALG-1 signal in all fractions) from Western blots of 2 independent experiments are shown (right). *P* value were obtained by two tailed *t*-test (**p value < 0.001).

To determine if ALG-1 was accumulating at the rough ER in *tbc-11(qbc24)* animals, we attempted to express the ER marker TRAM-1 in the animals’ seam cells but could not detect any protein expression in these cells. We therefore monitored the association of ALG-1 with polysomes in WT and *tbc-11* animals by polysome profiling. We fractionated worm extracts on a 15% to 55% sucrose gradient and then performed western blots for ALG-1 in the collected fractions. We did not observe any difference in ALG-1 distribution in *tbc-11*(*ok2576)* animals compared to wild-type (S5A Fig). However, we observed that in *tbc-11*(*qbc24)* animals, ALG-1 co-sediments more with the heavy polysomes fractions than in wild-type animals (Fig 5A; polysome profiles are shown in S5B Fig). To determine if ALG-1 was truly associating to polysomes, we repeated the fractionation after EDTA treatment to dissociate polysomes. The addition of EDTA successfully dissociated polysomes in both wild-type and *tbc-11(qbc24)* animals (S5C Fig). As expected in wild-type animals, ALG-1 shifted to fractions corresponding to low molecular weight complexes (Fig 5B). In contrast, we did not observe a shift as major for ALG-1 in *tbc-11(qbc24)* animal extracts treated with EDTA (Fig 5B). These results suggest that ALG-1 is not bound to polysomes in *tbc-11*(*qbc24)* animals but rather associates with an uncharacterized high molecular weight complex that is likely perinuclear, as observed by microscopy (Fig 4).

Altogether, our results show that the alteration of *tbc-11* induces an accumulation of unloaded ALG-1 in a perinuclear, high molecular weight complex that likely contains endomembranes (S6 Fig). This would prevent ALG-1 from binding to its mRNA targets and therefore induce miRNA-related defects in animals.

## Discussion

In this study, we uncovered a novel function for the RabGAP TBC-11 identified in an unbiased genetic screen that aimed to identify new genes involved in the microRNA pathway. Our work identified the small GTPase *rab-6* and the RabGAP *tbc-11* as important new actors in the gene regulation mediated by miRNAs in animals. We showed that *tbc-11* is likely a GAP for *rab-6* in *C*. *elegans* and that this small GTPase regulation is important for miRNA function. We demonstrated that in absence of *tbc-11*, the pool of miRNA-unloaded Argonaute ALG-1 that is likely associated to endomembranes increases, leading to a misregulation of miRNA targets. The alteration of TBC-11 function causes an accumulation of ALG-1 at the perinuclear region of the cell and forms an uncharacterized high molecular weight complex. We propose that this complex would sequester ALG-1, likely unbound to miRNA, and prevent its normally induced degradation. Further studies will be important to further characterize the nature of this complex.

Interestingly, this study represents our second forward genetic screens that identified new RAB-6-associated genes involved in the regulation of the miRNA pathway in *C*. *elegans*, highlighting the important role played by RAB-6 in the control of this gene regulatory pathway in animals [15]. Our work further supports previous reports suggesting that miRNA-mediated gene regulation is tightly linked to endomembrane trafficking. While we tend to limit our view of miRNA target repression to granules such as the P bodies, it is not so surprising to find ALG-1 associating to endomembranes such as the ER and Golgi. Indeed, the first report of Argonaute proteins described them as GERp95 (Golgi-endoplasmic reticulum protein 95 kDa) before knowing their precise function [42]. Several studies since then have linked the ER and Golgi to different steps of miRNA maturation and function. This physical sequestration could be important to allow coordination of miRISC loading, target repression, target decay and finally, Argonaute turnover. We also propose that intracellular trafficking could be important to repress mRNA targets at different subcellular locations within the cell. While it may seem surprising that a screen designed to be independent of miRNA loading allowed us to identify a gene implicated in intracellular trafficking, the proper localization of the λN tagged ALG-1 is likely still needed to allow repression of the targeted mRNA.

The activity of RAB-6 in the control of ALG-1 localization is likely reflected in our analysis of both mutant alleles of *tbc-11*. In animals where TBC-11 is deleted *(ok2576*), the activity of RAB-6 may be partially regulated by other GAPs with low affinity and thus the localization of ALG-1 is not as affected. While in *tbc-11(qbc24)* animals, a non-functional form of TBC-11 that likely retains its capacity to bind RAB-6 is expressed but unable to hydrolyze GTP, leading to a hyperactive form of RAB-6. While our results suggest that RAB-6 is important for the shuttling of ALG-1 to the endomembrane system, the nature of this interaction remains unknown. In fact, it is still to be determined if RAB-6 binds directly to ALG-1 or if another protein mediates this interaction. As RAB-6 interacts with the retrograde complex GARP, previously shown to be involved in the miRNA function in *C*. *elegans* by controlling the level of the GW182 protein AIN-1 and miRNAs [15], it is plausible that the GARP complex could be the mediator of the interaction between RAB-6 and ALG-1. However, despite several attempts, we have not been able to detect any proof of interaction between VPS-52 and ALG-1, even in *tbc-11(qbc24)* animals. Thus, we postulate that RAB-6 could affect the miRISC at numerous steps; its association with the GARP complex would affect the transport of AIN-1 and its association with a yet unknown effector protein would affect the transport of ALG-1.

While this study identified *rab-6* and the GAP *tbc-11* as factors controlling the localization of ALG-1 to a perinuclear complex, we propose that other Rabs are most likely involved in the proper localization of the Argonaute proteins. Indeed, different Rabs are responsible for precise steps of vesicular trafficking, and Argonaute proteins are likely transported within different compartments of the cell. We would therefore expect that other Rab proteins may affect the function of miRISC. Indeed, we observed that *rab-2* and *rab-14* RNAi induced alae breaks in wild-type animals, suggesting that they might also play a role in miRNA function. However, when RAB-6 is constitutively active, it excessively shuttles ALG-1 to the perinuclear region (see Model S6 Fig). This mis-shuttling leads to an accumulation of unloaded and unfunctional ALG-1.

These results highlight the importance of the regulation of Rab proteins for proper Argonaute function. Further studies will be essential to assess the role of other Rab proteins on the localization of Argonaute and other components of the miRNA pathway. Moreover, the processes occurring at different subcellular localization need to be clarified. Indeed, the subcellular localization at which miRISC loading, target repression and miRISC recycling occur remains poorly characterized, especially *in vivo*. Nevertheless, our results demonstrate that RAB-6 and TBC-11 are important in the proper localization of ALG-1 and that accumulation of unloaded ALG-1 at a perinuclear, high molecular weight complex impairs miRNA-mediated silencing in animals. Overall, this study emphasizes the importance of intracellular trafficking for miRNA function and proper miRISC localization.

## Methods

### Worm culture

*C*. *elegans* strains were cultured in standard conditions [43]. Strains were grown on Nematode Growth Media (NGM) plates and fed *E*. *coli* strain OP50. Worms were grown at 20°C unless specified.

### Strains used

N2 Bristol (WT), RB1959 *tbc-11(ok2576)*, MJS279 *tbc-11(qbc24)*, MT7626 *let-7 (n2853)*, MJS277 qbcSi11[*elt2p*::*GFP*::*his-58*::*3XmiR-228*::*tbb-2-3’UTR*] IV*; unc-119(ed3)III*, MJS276 qbcSi12[*elt-2p*::*GFP*::*his-58*::*3XmiR-228mut*::*tbb-2–3’UTR*] IV; *unc-119(ed3)III*, HML647 CRISPR tagged *GFP*::*alg-1*, MJS041 qbcSi03[*alg-1p*::*gfp*::*cog-1-boxb;cb-unc-119(+)*]IV; qbcSi05[*alg-1p*::*λN*::*mcherry*::*alg-1*::*alg-1 3’UTR;cb-unc-119(+)*] V, *612*.*5 col-10p*::*GFP*::*lin-41 3’UTR*

### Genetic screen and mutant identification

50 000 L4 staged MJS041 animals (*λN*::*mCherry*::*alg-1; GFP*::*box-B*) were mutagenized in 100 mM EMS for 4h. F2 animals were screened for GFP derepression. 19 candidates were isolated and named *tard-1* to *tard-19* (tethering *a**lg-1* repression defect). Candidate *tard-10(qbc24)* was mapped by crossing with a Hawaiian CB4856 strain in which *λN*::*mCherry*::*alg-1* and *gfp*::*box-B* constructs were inserted. Recombinants were sequenced and mutation was mapped as described in [44].

### RNA interference

RNAi was performed by feeding worms with IPTG inducible HT115 bacteria expressing dsRNA against *rab-6*.*1*, *rab-6*.*2*, *rab-2*, *rab-14*, *alg-1 or tbc-11* (List of oligonucleotides used to make RNAi constructs can be found in S1 Table and name of plasmids used in S2 Table). L1 staged animals were exposed to RNAi for 48 hours at 20°C and L4 or young adults were scored for either alae defects, ALG-1 localization or GFP expression.

### Preparation of protein extracts

Extracts were prepared from ~150,000 synchronized young adult animals. Worms were washed and resuspended in cold lysis buffer (10mM Potassium Acetate, 25mM Hepes-Potassium Hydroxide pH 7.0, 2mM Magnesium Acetate, 1mM DTT, 0.5% OR 1.5% [v/v] Triton X-100 and protease inhibitors) before being extracted with a Dounce homogenizer. The extracts were centrifuged at 17,000g for 20min at 4°C and the clarified supernatants were collected. For experiments comparing 0.5% vs 1.5% Triton, a single worm pellet was equally separated and extracted in parallel with different detergent concentrations.

### Western blotting

Extracts were quantified with Bio-Rad Protein Assay and equal amounts of proteins were resuspended in 2X SDS loading buffer (10mM Tris-HCl [pH 6.8], 2% [w/v] SDS, 100mM DTT and 10% [v/v] glycerol) before being heated at 95°C for 10 min. Samples were resolved onto an 8% polyacrylamide gel and transferred to 0,45um nitrocellulose blotting membranes (GE Healthcare). Membranes were incubated overnight at 4°C with the primary antibodies rabbit anti-ALG-1 diluted 1:1,000, rabbit anti-LIN-41 diluted 1:5,000, rabbit anti-AIN-1 diluted 1:5,000, mouse anti-beta-actin diluted 1:10,000 (List of Antibodies used in S3 Table). Membranes were then incubated with secondary antibody Sheep Anti-Mouse IgG or Goat Anti-Rabbit IgG.

### Immunoprecipitation and quantitative real-time PCR

Extracts were quantified with Bio-Rad Protein Assay and immuprecipitation (IP) was performed on 1mg of protein extract per condition. Anti-ALG-1 antibody was coupled to magnetic Dynabeads Protein G (ThermoFisher). Extracts were incubated with anti-ALG-1-coupled beads for 2h at 4°C. 10% of IP was loaded on polyacrylamide gel to assess ALG-1 levels. The remainder was submitted to RNA extraction by Trizol/Chloroform. The RNA was retrotranscribed with Multiscribe reverse transcriptase (ThermoFisher) and miRNA levels were measured with specific Taqman probes.

### Imaging and microscopy

DIC Nomarski images of worms’ alae, GFP fluorescence of miR-228 reporter [7] and hypodermal let-7 GFP microRNA reporter were taken with a Zeiss AxioCam HRm digital camera mounted on a Zeiss Axio Imager M1 microscope. Fluorescence intensity was measured with Zen software. Images of the GFP::ALG-1 intracellular location in the seam cells were taken with Zeiss LSM700 confocal microscope. Images were taken using the same settings and exposition time for each animal.

### Polysome profiles

Synchronized young adult animals were collected and washed in M9 supplemented with 1mM cycloheximide then washed once with lysis buffer (20mM Tris pH 8.5, 140 mM KCl, 1.5mM MgCl_2_, 1.5% [v/v] Nonidet P40, 1 mM DTT, 1 mM cycloheximide) before flash freezing. Thawed pellets were washed 3 times with lysis buffer and homogenized in 1 volume of lysis buffer supplemented with 0.4U/uL RNase using a Dounce homogenizer. For EDTA controls, a final concentration of 10mM of EDTA was added to the extracts following extraction. Concentration of RNA was measured with a spectrophotometer. 10 OD_260_ units were loaded onto a 15% to 55% sucrose gradient and further fractionated and analyzed as described in [45]. For EDTA controls, MgCl_2_ and cycloheximide in sucrose gradients were replaced by EDTA. Recovered fractions were precipitated with 2 volumes of ethanol 100% and 10% of each fraction was loaded on an 8% SDS-polyacrylamide gel for western blot analysis.

### miRNA cloning, sequencing and analysis

The TruSeq small RNA libraries were prepared and sequenced as described in [46,47]. The small RNA sequencing data are available at Gene Expression Omnibus (GEO) with the accession number: GSE141719.

Sequencing reads were mapped to the genome and cDNA using custom PERL (5.10.1) scripts and Bowtie 0.12.7 [48]. Databases used include *C*. *elegans* genome (WormBase release WS215), Repbase 15.10 [49], and miRBase 16 [50]. The Generic Genome Browser [51] was used to visualize the alignments. Detailed PERL scripts and related database files and analyses in this study are available upon request.

The samples were normalized to the total small RNAs including miRNAs, 22G-RNAs and 21U-RNAs. We used the average of triplicates for each strain to calculate the ratio of each small RNA reads of *tbc-11* to the sum of *tbc-11* + wild-type reads.

### Figure data

Data for main text Figs 1–3 and 5 and S1 and S2 Figs can be found in S1 File.

## Supporting information

S1 Fig Characterization of *tbc-11(qbc24)* and *tbc-11(ok2576)* mutant alleles. **(A)** GFP reporter repressed by λN::mCherry tagged ALG-1. GFP was fused to *alg-1* endogenous promoter (*alg-1p*) and *cog-1* 3’ UTR in which the *lsy-6* binding sites were replaced by 6 box B sequences (cog-1Δlsy-6 bs). (**B**) DIC and fluorescent microscopy of WT and *tbc-11(qbc24)* pharynx in young adults. GFP is derepressed in *tbc-11(qbc24)* animals. ALG-1 expression (represented by mCherry) is not affected in these animals. Images were taken using the same settings and exposition time for each animal. Scale bar: 50μm. (**C**) Percentage of alae breaks of wild-type (WT), *tbc-11(qbc24)* homozygous, and *tbc-11(qbc24)* heterozygous (*tbc-11(qbc24*)/+) young adult animals. *tbc-11(qbc24)* heterozygous animals were observed as F1 of a genetic cross between *tbc-11(qbc24)* and wild-type animals. *P* value were obtained by one-way ANOVA (**p value < 0.001) (**D**) Number of seam cells in *tbc-11(qbc24)* and *(ok2576)* alleles. Strains were crossed with a strain expressing GFP in the seam cells (*scm*::GFP) in order to score them. Animals were observed as young adults. Wild-type (WT) animals have an invariable number of 16 seam cells. *P* value were obtained by Fisher’s exact test (***p value < 0.0001) (**E**) Left: DIC and fluorescent microscopy of *col-10*::*gfp*::*lin-41* 3’UTR reporter in hypodermal cells of L4 staged animals. Animals were fed with bacteria expressing RNAi against *tbc-11* or control RNAi (no targeting gene) for 48 hours. Scale bar: 50μm. Right: Quantification of GFP fluorescence intensity in four hypodermal cells per animal. Images were taken using the same settings and exposition time for each animal. The number of animals scored (n) is indicated. *P* value were obtained by two tailed t-test. (*p value < 0.05). **(F)** Left: DIC and fluorescent microscopy of miR-228 mutated reporter in intestine cells of L2 staged animals. Scale bar: 50μm. Right: Quantification of GFP fluorescence intensity in four intestine cells per animal. Images were taken using the same settings and exposition time for each animal. The number of animals scored (n) is indicated. *P* value were obtained by two tailed t-test. ns: non-significant.
(TIF)

S2 Fig Effect of *rab-2* and *rab-14* RNAi on alae structure. Alae breaks of *tbc-11(ok2576)* were scored under DIC Nomarski microscopy. Animals were fed with bacteria expressing RNAi against *rab-2* (upper panel), *rab-14* (lower panel) or control RNAi (no targeting gene) for 48 hours and observed as young adults. 50 animals were observed for each condition. Each circle represents the mean of one independent RNAi experiment. *P* value were obtained by one-way ANOVA (***p value < 0.0001).
(TIF)

S3 Fig Protein levels of ALG-1 and AIN-1 in *tbc-11* animals and quantification of microRNA levels. **(A)** Western blot of ALG-1 and the GW182 protein AIN-1 in wild-type (WT), *tbc-11(qbc24)* and *tbc-11(ok2576)* young adult animals. Extracts were prepared with standard conditions (0.5% triton). Actin is used as a loading control. **(B)** Western blot of the GW182 protein AIN-1 detected in wild-type (WT), *tbc-11(qbc24)* and *tbc-11(ok2576)* young adult worms extracts prepared with low (0.5% triton) or high (1.5% triton) detergent concentration. High detergent concentration allows better extraction of membrane associated proteins. Actin is used as a loading control. **(C)** Western blot of ALG-1 protein in wild-type (WT), *tbc-11 (qbc24)* and *tbc-11(ok2576)*. Proteins were fully solubilized by boiling animals in Laemmli buffer for 10 minutes. Actin is used as a loading control. **(D)** Small RNA sequencing of *tbc-11(qbc24)* (left) and *tbc-11(ok2576)* (right) young adult animals compared to wild-type animals. The dotted line represents a two-fold change. The number of miRNA analyzed is indicated (n). *p*<0.22 for let-7 in *tbc-11(qbc24)*, *p*<0.50 for miR-228 in *tbc-11(qbc24)*, *p*<0.13 for let-7 in *tbc-11(ok2576)*, *p*<0.25 for miR-228 in *tbc-11(ok2576)*. *P* value for individual miRNAs were obtained by two tailed unpaired t test. The samples were normalized to the total small RNAs including miRNAs, 22G-RNAs and 21U-RNAs. **(E)** Representative western blot of immunoprecipitations of ALG-1 quantified in Fig 3C.
(TIF)

S4 Fig Co-localization of ALG-1 and the Golgi marker MANS. DIC and fluorescent microscopy of intracellular localization of endogenously tagged GFP::ALG-1 in seam cells of wild-type (WT; **A**) and *tbc-11(qbc24)* (**B**) animals. Animals are expressing a plasmid containing the sequence of *alg-1p*::mCherry::MANS as a marker of the Golgi. Merge image represents the overlap of GFP (ALG-1), mCherry (MANS) and DIC images. The nucleus of the seam cell is indicated by an arrow. Zoomed in images of GFP::ALG-1 merged with mCherry::MANS in a seam cell are shown in insets. Images were taken using the same settings and exposition time for each animal. Scale bar: 10 μm.
(TIF)

S5 Fig Polysome profiles and quantification of *let-7* in fractions. (**A**) Top: Polysome profiles of wild-type (WT) and *tbc-11(ok2576)* young adult animals performed with extracts prepared with high detergent concentration (1.5% triton). Bottom: Detection of ALG-1 in different fractions by Western blot. Polysome profiles show no differences in overall translation. Low and high exposure of the same membrane are shown. (**B**) Polysome profiles for wild-type (WT) and *tbc-11(qbc24)* extracts presented in Fig 5A. Fractions corresponding to ribosomal subunits, light polysomes and heavy polysomes are indicated. **(C)** Polysome profiles for wild-type (WT) and *tbc-11(qbc24)* extracts treated with EDTA presented in Fig 5B. Fractions corresponding to ribosomal subunits, light polysomes and heavy polysomes are indicated.
(TIF)

S6 Fig Model for the implication of RAB-6 and TBC-11 in transport of ALG-1. In absence of TBC-11, RAB-6 is constitutively active and shuttles miRNA-bound and unbound ALG-1 to the perinuclear region.
(TIF)

S1 Table List of oligonucleotides primers used in this study. (DOCX)

S2 Table List of plasmids used in this study. (DOCX)

S3 Table List of antibodies used in this study. (DOCX)

S1 File Data tables for main text Figs 1–3 and 5 and S1 and S2 Figs. (XLSX)
