# Supplementary figures and images for: The RabGAP TBC-11 controls Argonaute localization for proper microRNA function in C. elegans
Source: PLoS Genet. 2021 Apr 7;17(4):e1009511. doi: 10.1371/journal.pgen.1009511 (PMC8055011; doi:10.1371/journal.pgen.1009511)

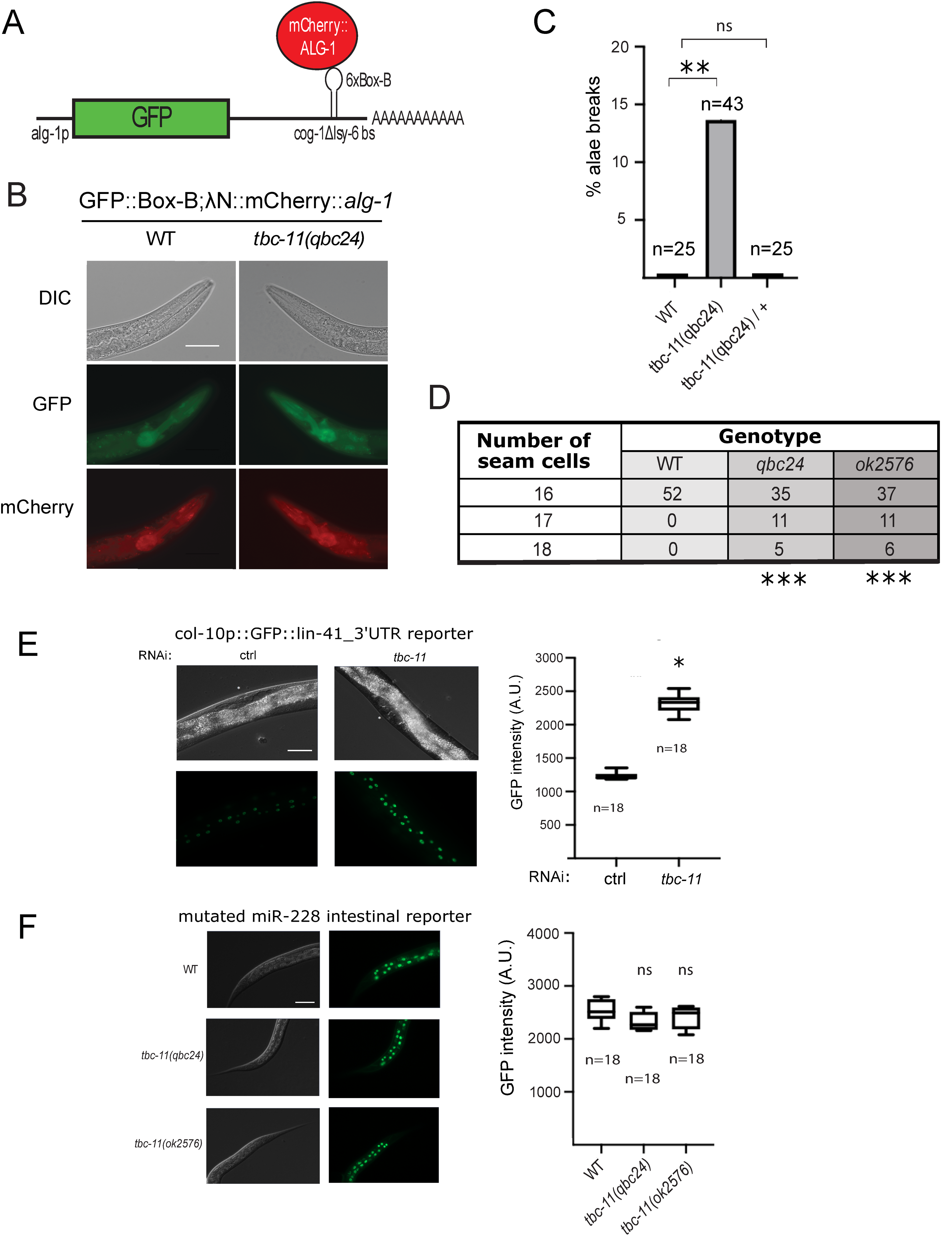

Supplement: S1 Fig — (A) GFP reporter repressed by λN::mCherry tagged ALG-1. GFP was fused to alg-1 endogenous promoter (alg-1p) and cog-1 3’ UTR in which the lsy-6 binding sites were replaced by 6 box B sequences (cog-1Δlsy-6 bs). (B) DIC and fluorescent microscopy of WT and tbc-11(qbc24) pharynx in young adults. GFP is derepressed in tbc-11(qbc24) animals. ALG-1 expression (represented by mCherry) is not affected in these animals. Images were taken using the same settings and exposition time for each animal. Scale bar: 50μm. (C) Percentage of alae breaks of wild-type (WT), tbc-11(qbc24) homozygous, and tbc-11(qbc24) heterozygous (tbc-11(qbc24)/+) young adult animals. tbc-11(qbc24) heterozygous animals were observed as F1 of a genetic cross between tbc-11(qbc24) and wild-type animals. P value were obtained by one-way ANOVA (**p value < 0.001) (D) Number of seam cells in tbc-11(qbc24) and (ok2576) alleles. Strains were crossed with a strain expressing GFP in the seam cells (scm::GFP) in order to score them. Animals were observed as young adults. Wild-type (WT) animals have an invariable number of 16 seam cells. P value were obtained by Fisher’s exact test (***p value < 0.0001) (E) Left: DIC and fluorescent microscopy of col-10::gfp::lin-41 3’UTR reporter in hypodermal cells of L4 staged animals. Animals were fed with bacteria expressing RNAi against tbc-11 or control RNAi (no targeting gene) for 48 hours. Scale bar: 50μm. Right: Quantification of GFP fluorescence intensity in four hypodermal cells per animal. Images were taken using the same settings and exposition time for each animal. The number of animals scored (n) is indicated. P value were obtained by two tailed t-test. (*p value < 0.05). (F) Left: DIC and fluorescent microscopy of miR-228 mutated reporter in intestine cells of L2 staged animals. Scale bar: 50μm. Right: Quantification of GFP fluorescence intensity in four intestine cells per animal. Images were taken using the same settings and exposition time for each animal. The [file pgen.1009511.s001.tif]

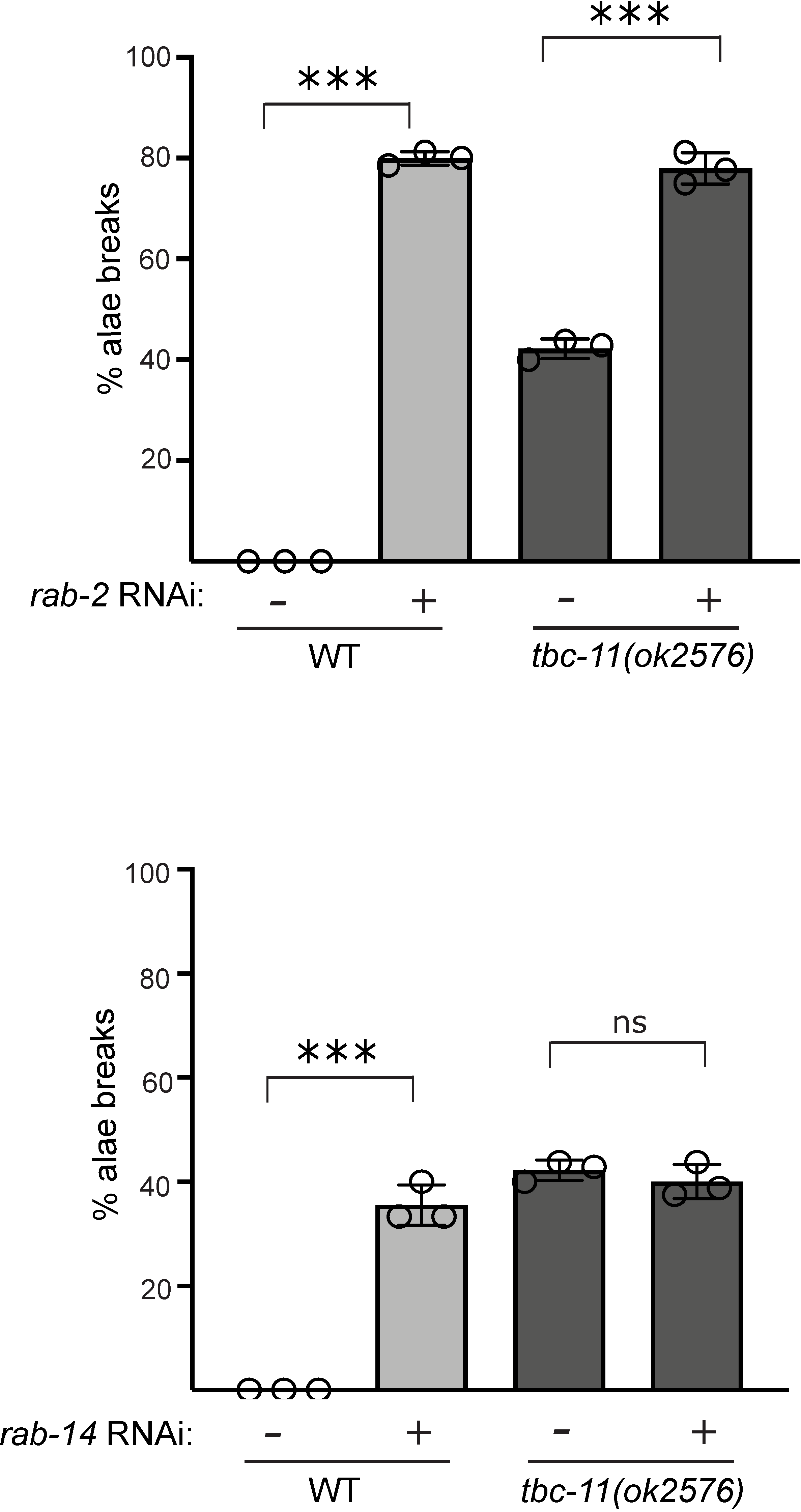

Supplement: S2 Fig — Alae breaks of tbc-11(ok2576) were scored under DIC Nomarski microscopy. Animals were fed with bacteria expressing RNAi against rab-2 (upper panel), rab-14 (lower panel) or control RNAi (no targeting gene) for 48 hours and observed as young adults. 50 animals were observed for each condition. Each circle represents the mean of one independent RNAi experiment. P value were obtained by one-way ANOVA (***p value < 0.0001). (TIF) [file pgen.1009511.s002.tif]

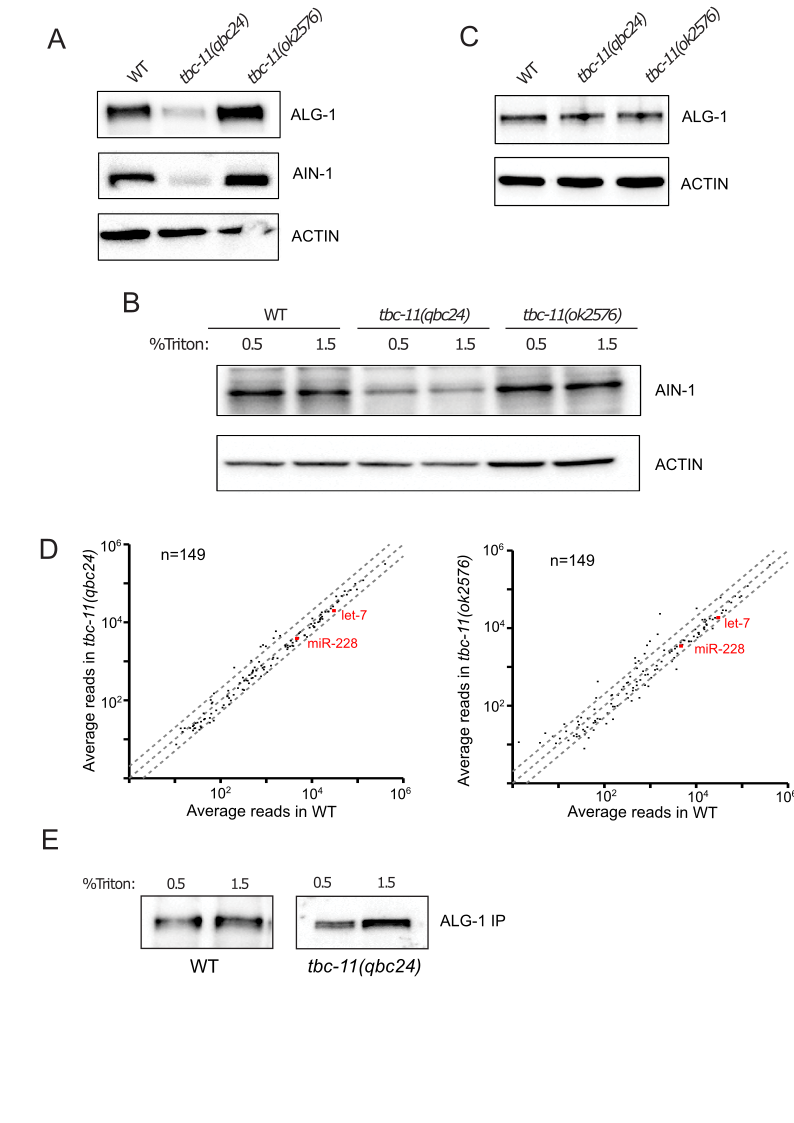

Supplement: S3 Fig — (A) Western blot of ALG-1 and the GW182 protein AIN-1 in wild-type (WT), tbc-11(qbc24) and tbc-11(ok2576) young adult animals. Extracts were prepared with standard conditions (0.5% triton). Actin is used as a loading control. (B) Western blot of the GW182 protein AIN-1 detected in wild-type (WT), tbc-11(qbc24) and tbc-11(ok2576) young adult worms extracts prepared with low (0.5% triton) or high (1.5% triton) detergent concentration. High detergent concentration allows better extraction of membrane associated proteins. Actin is used as a loading control. (C) Western blot of ALG-1 protein in wild-type (WT), tbc-11 (qbc24) and tbc-11(ok2576). Proteins were fully solubilized by boiling animals in Laemmli buffer for 10 minutes. Actin is used as a loading control. (D) Small RNA sequencing of tbc-11(qbc24) (left) and tbc-11(ok2576) (right) young adult animals compared to wild-type animals. The dotted line represents a two-fold change. The number of miRNA analyzed is indicated (n). p<0.22 for let-7 in tbc-11(qbc24), p<0.50 for miR-228 in tbc-11(qbc24), p<0.13 for let-7 in tbc-11(ok2576), p<0.25 for miR-228 in tbc-11(ok2576). P value for individual miRNAs were obtained by two tailed unpaired t test. The samples were normalized to the total small RNAs including miRNAs, 22G-RNAs and 21U-RNAs. (E) Representative western blot of immunoprecipitations of ALG-1 quantified in Fig 3C. (TIF) [file pgen.1009511.s003.tif]

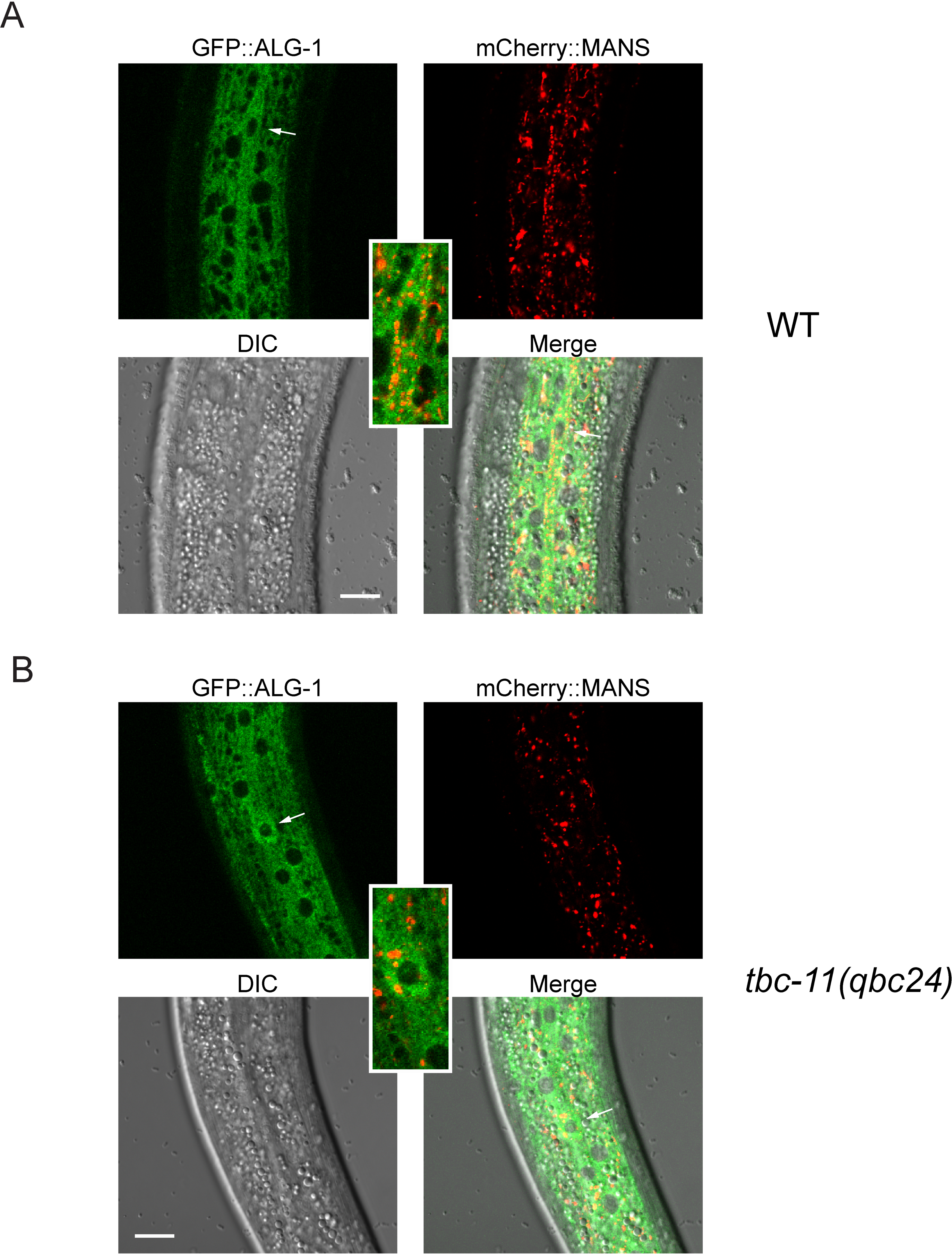

Supplement: S4 Fig — DIC and fluorescent microscopy of intracellular localization of endogenously tagged GFP::ALG-1 in seam cells of wild-type (WT; A) and tbc-11(qbc24) (B) animals. Animals are expressing a plasmid containing the sequence of alg-1p::mCherry::MANS as a marker of the Golgi. Merge image represents the overlap of GFP (ALG-1), mCherry (MANS) and DIC images. The nucleus of the seam cell is indicated by an arrow. Zoomed in images of GFP::ALG-1 merged with mCherry::MANS in a seam cell are shown in insets. Images were taken using the same settings and exposition time for each animal. Scale bar: 10 μm. (TIF) [file pgen.1009511.s004.tif]

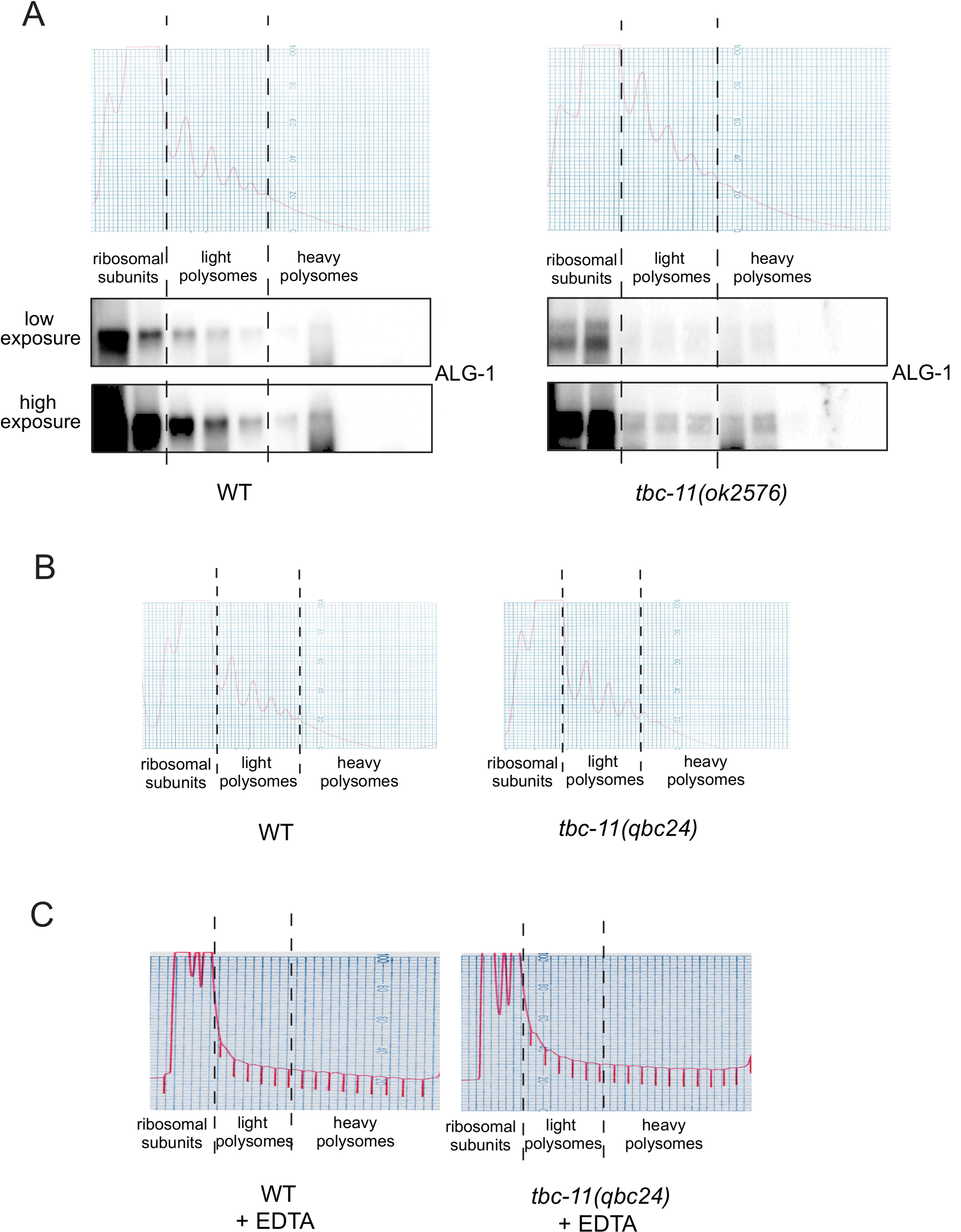

Supplement: S5 Fig — (A) Top: Polysome profiles of wild-type (WT) and tbc-11(ok2576) young adult animals performed with extracts prepared with high detergent concentration (1.5% triton). Bottom: Detection of ALG-1 in different fractions by Western blot. Polysome profiles show no differences in overall translation. Low and high exposure of the same membrane are shown. (B) Polysome profiles for wild-type (WT) and tbc-11(qbc24) extracts presented in Fig 5A. Fractions corresponding to ribosomal subunits, light polysomes and heavy polysomes are indicated. (C) Polysome profiles for wild-type (WT) and tbc-11(qbc24) extracts treated with EDTA presented in Fig 5B. Fractions corresponding to ribosomal subunits, light polysomes and heavy polysomes are indicated. (TIF) [file pgen.1009511.s005.tif]

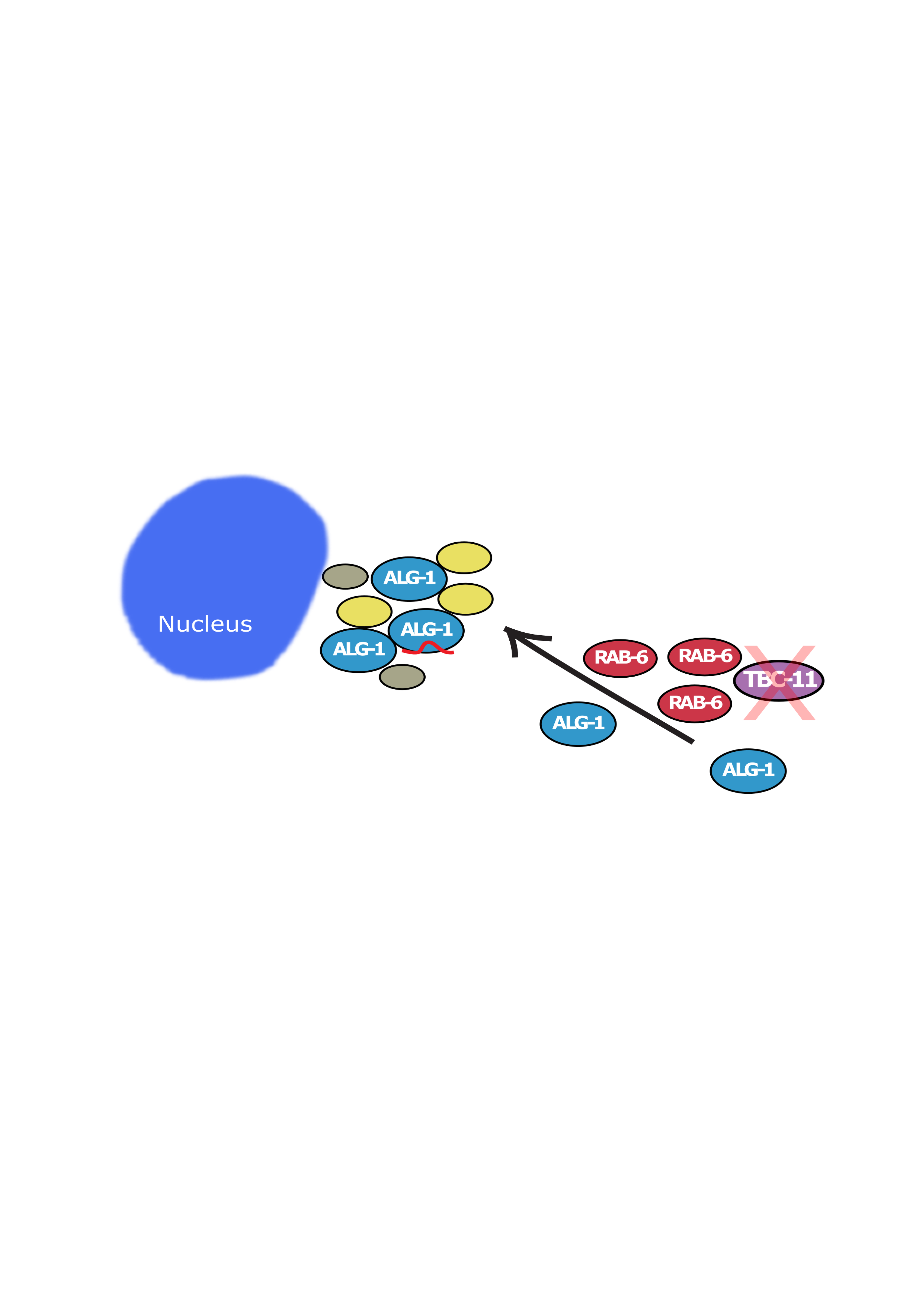

Supplement: S6 Fig — In absence of TBC-11, RAB-6 is constitutively active and shuttles miRNA-bound and unbound ALG-1 to the perinuclear region. (TIF) [file pgen.1009511.s006.tif]
