# Supplementary material for: The RabGAP TBC-11 controls Argonaute localization for proper microRNA function in C. elegans
Source: PLoS Genet. 2021 Apr 7;17(4):e1009511. doi: 10.1371/journal.pgen.1009511 (PMC8055011; doi:10.1371/journal.pgen.1009511)
Supplement: S1 Table — (DOCX) [file pgen.1009511.s007.docx]

**S1 Table. List of oligonucleotides primers used in this study**

| Primers | Insert to amplify | Sequence |
| --- | --- | --- |
| Mso2847 | *rab-6.1*, adds XbaI | 5’-agttctagaGAAACTTCTGCAAAAGCTGGC-3’ |
| Mso2848 | *rab-6.1*, adds KpnI | 5’-gtaggtaccTTAACACGGACATTGACGGC-3’ |
| Mso2815 | *rab-6.2*, adds XbaI | 5’-agttctagaGAACGTTTCCGCTCACTCATC-3’ |
| Mso2816 | *rab-6.2*, adds KpnI | 5’-gtaggtaccACATTAAGCTCCTTGGCCTTTC-3’ |
| Mso2986 | *tbc-11,* adds XbaI | 5’-agttctagaAAGAAATTGGCCGTCACCAAG-3’ |
| Mso2987 | *tbc-11,* adds KpnI | 5’-gtaggtaccATAATCGCCTTCTTCTGCACC-3’ |
| Mso3452 | *rab-2*, adds SacI | 5’-taagcagagctcACATCTTGGCTTGAGGATGC-3’ |
| Mso3453 | *rab-2*, adds KpnI | 5’-taagcaggtaccTAACAGCATCCAGATCCACC-3’ |
| Mso3454 | *rab-14*, adds SacI | 5’-taagcagagctcATTGGTGATATGGGTGTCGG-3’ |
| Mso3455 | *rab-14*, adds KpnI | 5’-taagcaggtaccGCACGTCACGTTGATCTTCG-3’ |
