# Supplementary material for: The RabGAP TBC-11 controls Argonaute localization for proper microRNA function in C. elegans
Source: PLoS Genet. 2021 Apr 7;17(4):e1009511. doi: 10.1371/journal.pgen.1009511 (PMC8055011; doi:10.1371/journal.pgen.1009511)
Supplement: S2 Table — (DOCX) [file pgen.1009511.s008.docx]

**S2 Table. List of plasmids used in this study**

| Plasmid | Backbone | Insert |
| --- | --- | --- |
| Msp0411 | L4440 | *rab-6.1* |
| Msp0413 | L4440 | *rab-6.2* |
| Msp0429 | L4440 | *rab-2* |
| Msp0431 | L4440 | *rab-14* |
| Msp0171* | L4440 | *alg-1* |
| Msp0418 | pPV549 | *alg-1p::MANS::mCherry* |
| Msp0414 | L4440 | *tbc-11* |

*****see [1]

References:

1. Bouasker S, Simard MJ. The slicing activity of miRNA-specific Argonautes is essential for the miRNA pathway in C. elegans. Nucleic Acids Res. 2012;40(20):10452-62.
