# Supplementary material for: The RabGAP TBC-11 controls Argonaute localization for proper microRNA function in C. elegans
Source: PLoS Genet. 2021 Apr 7;17(4):e1009511. doi: 10.1371/journal.pgen.1009511 (PMC8055011; doi:10.1371/journal.pgen.1009511)
Supplement: S3 Table — (DOCX) [file pgen.1009511.s009.docx]

**S3 Table. List of antibodies used in this study**

| Antibody | Source | Identifier |
| --- | --- | --- |
| Rabbit anti-AIN1 polyclonal antibody | Custom made | For info, see [1] |
| Rabbit anti-ALG1 Polyclonal antibody | Custom made | For info, see [2] |
| Mouse anti-beta Actin Monoclonal antibody | Abcam | Cat# ab49900 |
| Rabbit anti-LIN41 Polyclonal antibody | Chris Hammel | - |
| Peroxidase-AffiniPure Sheep Anti-Mouse IgG | Jackson Immunoresearch labs | Cat#515-035-062 |
| Peroxidase-AffiniPure Goat Anti-Rabbit IgG | Jackson Immunoresearch labs | Cat#111-035-144 |

References:

1. Jannot G, Michaud P, Quevillon Huberdeau M, Morel-Berryman L, Brackbill JA, Piquet S, et al. GW182-Free microRNA Silencing Complex Controls Post-transcriptional Gene Expression during Caenorhabditis elegans Embryogenesis. PLoS Genet. 2016;12(12):e1006484.

2. Bukhari SI, Vasquez-Rifo A, Gagne D, Paquet ER, Zetka M, Robert C, et al. The microRNA pathway controls germ cell proliferation and differentiation in C. elegans. Cell Res. 2012;22(6):1034-45.
